# Disruption of *Foxg1* impairs neural plasticity leading to social and cognitive behavioral defects

**DOI:** 10.1186/s13041-019-0484-x

**Published:** 2019-06-28

**Authors:** Baocong Yu, Junhua Liu, Mingzhao Su, Chunlian Wang, Huanxin Chen, Chunjie Zhao

**Affiliations:** 1 Key Laboratory of Developmental Genes and Human Diseases, MOE, School of Medicine, Southeast University, Nanjing, 210009 China; 2 Key Lab of Cognition and Personality, MOE, School of Psychology, Southwest University, Chongqing, 400715 China

**Keywords:** Neural plasticity, Dendritic arborization, Spine, Spatial learning and memory, *Foxg1*, *FOXG1* syndrome, LTP

## Abstract

The transcription factor *Foxg1* is known to be continuously expressed at a high level in mature neurons in the telencephalon, but little is known about its role in neural plasticity. Mutations in human *FOXG1* cause deficiencies in learning and memory and limit social ability, which is defined as *FOXG1* syndrome, but its pathogenic mechanism remains unclear. To examine the role of *Foxg1* in adults, we crossed *Camk2a-Cre*^*ER*^ with *Foxg1*^*fl/fl*^ mice and conditionally disrupted *Foxg1* with tamoxifen in mature neurons. We found that spatial learning and memory were significantly impaired when examined by the Morris water maze test. The cKO mice also showed a significant reduction in freezing time during the contextual and cued fear conditioning test, indicating that fear conditioning memory was affected. A remarkable reduction in Schaffer-collateral long-term potentiation was also recorded. Morphologically, the dendritic arborization and spine densities of hippocampal pyramidal neurons were significantly reduced. Primary cell culture further confirmed altered dendritic complexity after *Foxg1* deletion. Our results indicated that *Foxg1* plays an important role in maintaining the neural plasticity, which is vital to high-grade function.

## Introduction

Most neurons have several dendrites that function as information inputs and single axons that serve as information outputs [1]. During development, primary dendrites are derived from the cell body and branch to form complicated secondary and tertiary dendrites [2]. However, dendrite arbors are highly dynamic, extending and retracting branches as maturation proceeds. The size and shape of a neuron’s dendrite arbor are critical for the determination of the number and distribution of receptive synaptic contacts [3]. Spines are small protrusions that are derived from dendrites and harbor the majority of glutamatergic excitatory synapses in the mammalian forebrain [4, 5]. Dendritic spines undergo experience-dependent morphological changes, and even subtle changes in dendritic spines may have marked effects on synaptic function, plasticity and patterns of connectivity in neuronal circuits. Changes in dendritic spine number and morphology are accompanied with the formation, maintenance and elimination of synapses and facilitate the establishment and remodeling of connectivity within neuronal circuits [4]. In adulthood, most dendrite arbors and dendritic spines are stable for months, years and possibly even decades [3]. Emerging evidence has revealed that losses of dendritic spine and dendrite arbor stability are major contributing factors to the impaired perceptive, cognitive and social ability observed in a variety of neuropsychiatric disorders, such as schizophrenia and autism disease (ASD) [3, 4, 6, 7]. However, the molecular and cellular basis underlying neural plasticity requires further exploration.

*Foxg1,* a member of the Forkhead box transcription factor family, has been reported to be critical for telencephalon development [8]. Early disruption of *Foxg1* results in severe defects in pattern formation, cell proliferation, cell fate determination and neuronal migration [9–16]. Mutations in *Foxg1* cause a severe neurodevelopmental disorder known as *FOXG1* syndrome (former cognition variant of Rett syndrome) [17]. Patients suffer from seizure, mental retardation, and social and cognitive disabilities [18–21]. Considering that strong expression of *Foxg1* persists into adulthood [22], *Foxg1* may play an import role in neural plasticity as well. Recently, via overexpression and knockdown of *Foxg1,* Chiola et al. demonstrated that *Foxg1* promotes neurite elongation in postmitotic neurons [23]. Here, by crossing *Camk2a-Cre*^*ER*^ with *Foxg1*^*fl/fl*^ lines combined with tamoxifen induction, FOXG1 was conditionally disrupted in mature neurons in adult. We found a severe reduction in dendritic complexity and spine density accompanied with decreased hippocampal long-term potentiation (LTP). The cKO mice exhibited deficiencies in learning and memory and limited social abilities. This study revealed a new role for *Foxg1* in the maintenance of neural plasticity.

## Materials and methods

### Animals

The *Foxg1*^*fl/fl*^ line was generated as previously described [11]. *Camk2a-Cre*^*ER*^ (stock number: 012362) and *Rosa-YFP* (stock number: 006148) mice were purchased from the Jackson Laboratory. *Nex-Cre* mice were previously described [24]. Mice for behavioral tests and electrophysiological recording were maintained on a C57BL6J background, and on an ICR background for histological analysis. The day of birth was defined as postnatal day 0 (P0). The *Camk2a-Cre*^*ER*^*;Foxg1*^*fl/fl*^ mice were referred to as *Foxg1* conditional knockout (cKO), and the *Foxg1*^*fl/fl*^ mice were referred to as wild-type (WT). For tamoxifen (TM) induction, mice were given an intraperitoneal injection of TM at 100 μg/g body weight once a day for 5 consecutive days around P60. Thirty days later, mice were processed as described below. For histological analysis, gender was not considered. For behavioral tests, only male mice were used. All animals were housed in the animal facility of Southeast University, and all experimental procedures followed the guidance approved by Southeast University.

### Immunostaining and cell counting

Brains were perfused and post-fixed with 4% paraformaldehyde at 4 °C for 12–16 h, cryoprotected in 30% sucrose and embedded in optimum cutting temperature compound (OCT). Coronal sections (20 μm thick) were obtained using a Leica cryostat (CM 3050S). Immunostaining was performed as previously described [11]. Chicken anti-GFP (Abcam, ab13970, 1:500) and rabbit anti-FOXG1 (Abcam, AB18259, 1:500) were used as primary antibodies, and Alexa Fluor 488 goat anti-chicken IgG (Molecular Probes, A11039, 1:500) and Alexa Fluor 546 donkey anti-rabbit IgG(Molecular Probes, A10040, 1:500) were used as secondary antibodies. DAPI (Sigma, D9564, 1:1000) was incubated for 15 min (mins) before coverslips were applied. Images were captured by a confocal microscope (Olympus, FV1000). For cell counting, three brains from each genotype from at least two different litters were collected and images of two consecutive hippocampal slices of each brain were used; the dorsal hippocampus was outlined using ImageJ software (NIH), and CA1 cell numbers were counted manually based on the DAPI staining.

### Quantitative real-time polymerase chain reaction (qRT-PCR)

qRT-PCR was performed as previously described [25]. Hippocampi, dorsal cortex and amygdala from at least three brains of each genotype were used. The primers used were: *Foxg1*, 5′-TGGCAACACTGCCCATTCA-3′ and 5′- CATTTGCGCAACACAGGTTA-3′; *Gapdh*, 5′-ACCCACTCCTCCACCTTTGAC-3′ and 5′-TGTTGCTGTAGCCAAATTCGTT- 3′. *Foxg1* mRNA expression levels were normalized to the corresponding *Gapdh* mRNA levels. An unpaired Student’s *t-*test was used to determine the significance.

### Western blot

Hippocampi were collected and prepared as described previously [11]. The primary antibodies were: rabbit anti-FOXG1 (Abcam, AB18259, 1:2000), rabbit anti-GAPDH (Cell Signaling Technology, 5174S, 1:3000); the secondary antibody was HRP-linked anti-rabbit IgG (Cell Signaling Technology, 7074S, 1:3000). Densitometric analysis was performed using ImageJ software, and the intensity of each band was normalized to the intensity of the corresponding GAPDH band. An unpaired Student’s *t-*test was used to determine the significance.

### Behavioral tests

Behavioral tests were performed using male mice in the following order: open-field test, elevated O-maze, social behavior test, Morris water maze and contextual and cued fear conditioning test with an interval of 3 days between each test. All tests were conducted during the light phase. All videos were taken using high-resolution digital cameras and analyzed by EthoVision software (Noldus), except for the contextual and cued fear conditioning test, which was analyzed manually in a double-blinded manner.

#### Open-field test

For the open-field test, each mouse was placed in a 40 × 40 cm square chamber as previously described [26]. Data were collected for 30 mins. Average velocity and total distance moved were measured to assess locomotor and exploratory activity, whereas the proportion of time spent in the center of the enclosure in the first 5 mins was used as a measure of anxiety.

Elevated O-maze test:

The O-maze equipment was a 100 cm in height, 45 cm in outer diameter and 6 cm wide ring with two open arms and two closed arms. Mice were placed at the open-closed boundary facing the open side. The total recording time was 10 mins, and the time spent in either the open or closed arms was measured.

#### Social behavior test

The three-chamber box contained three 20 × 40 cm chambers with open middle sections on each of the transparent dividing walls. The test was divided into three phases. In the first phase, mice were placed in the middle of the three-chamber box and allowed to habituate for 5 mins. In the second phase, two cup-like cages were put into the left and right side of the chamber box with one caged a pre-trained male mouse. The placement of the caged mouse in the left or right side of the chamber was systematically altered between trials. Mice were placed in the middle and recorded for 10 mins. In the last phase, the empty cage in the second phase was caged with a new pre-trained male mouse, and mice were allowed to explore for 10 mins. The exploration time in each side of the three-chamber box was measured during the three phases.

#### Morris water maze

The Morris water maze was used to access spatial learning and memory [27]. The maze was a circular pool with a diameter of 120 cm and a height of 50 cm. It was divided into four equal quadrants (NW, NE, SW and SE) and filled with whitening water with a temperature of 20–22 °C. Mice were trained four times a day to find a hidden platform in the SW quadrant by using extra cues. Mice that failed to locate the platform within 60 s were led to the platform to stay for 15 s. The training sessions lasted for 8 days. The probe test was conducted 24 h after the training session. The platform was removed and mice were placed into the NE quadrant and allowed to explore for 60 s. The daily average latency to reach the platform for each mouse was used to assess learning progress. The time spent in each quadrant and the frequency of entry on the platform zone during the probe test were analyzed to evaluate spatial memory.

#### Contextual and cued fear conditioning test

To assess fear memory, mice were placed into the apparatus (Ugo Basile) and allowed to habituate for 180 s, then the mice were given an auditory cue (2800 HZ) for 30 s, followed by a 0.2 mA foot shock at the last 1 s. The tone-shock procedure was repeated three times. The contextual fear test was conducted 24 h after the training session. Mice were placed into the chamber and recorded for 8 mins. The cued fear test was conducted on the next day. Mice were placed into a new testing chamber for 3 mins and then given the auditory cue used in the training session for 8 mins. The cued fear memory recall was conducted 7 days later by using the same procedures in the cued fear test. The freezing time during each period was analyzed.

### Electrophysiology

#### Slice preparation

Mice were anesthetized by inhalation of isoflurane and decapitated; the brains were rapidly removed and immersed in ice-cold artificial cerebrospinal fluid (ACSF) containing (mM) 185 sucrose, 20 D-glucose, 2.5 KCl, 1.25 NaH_2_PO_4_, 26 NaHCO_3_, 1 CaCl_2_, 6 MgCl_2_ and saturated with 95% O_2_ and 5% CO_2_ (pH 7.4). Transverse hippocampal slices were cut using a vibrating microtome (Leica Microsystems, VT1000s) at a thickness of 350 μm. Slices were recovered at 32 °C for 30 mins in the same ACSF but with sucrose replaced by 124 mM NaCl, and then maintained at room temperature (22 °C) for at least 1 h before recording.

#### Electrophysiological measurements

Slices were transferred to a submerged recording chamber with continuously perfused (2 ml/min, room temperature) ACSF containing (mM) 124 NaCl, 20 D-glucose, 2.5 KCl, 1.25 NaH_2_PO_4_, 26 NaHCO_3_, 4 CaCl_2_, 4 MgCl_2_, and 50 μM picrotoxin (Sigma, R284556) to block GABAergic synaptic activity. Whole-cell recordings were performed from CA1 pyramidal cells in voltage-clamp mode using a Heka EPC10 amplifier (Heka Instruments); cells were voltage-clamped at − 70 mV. For the cKO cell recording, YFP^+^ cells were first identified under fluorescence and then recorded under illumination differential interference contrast videomicroscopy. Recording electrodes (3–5 MΩ) were filled with internal solution containing (mM) 125 CsCl, 8 NaCl, 10 HEPES, 2 MgATP, 0.3 NaGTP, 0.2 EGTA and 0.1% biocytin (Sigma, B4261) (pH =7.4). Spontaneous excitatory postsynaptic current (sEPSC) was first recorded for 8 min and analyzed in Mini Analysis (Synaptosoft) with event detection levels for synaptic currents set at 5 pA. All the events were checked manually afterwards. Excitatory postsynaptic current (EPSC) was evoked by glass electrodes filled with ACSF. Stimulation was given by a stimulus isolator (ISO-Flex) at an intensity that evoked 50% of the maximal EPSC response, with a duration of 0.1 ms and a frequency of 0.1 Hz. Long-term potentiation (LTP) was induced by a pairing protocol that contained 200 pulses at 1.4 Hz paired with a long depolarization (~ 3 min to 0 mV). Series and input resistances during the recording were monitored every 10 s by measuring the peak and steady-state currents in response to 4 mV, 40 ms depolarizing pulses. Recording was conducted for 10 mins for baseline and 40 mins after induction. Only the cells with series resistance under 20 MΩ and with a variation in series resistance under 25% were analyzed. LTP was measured by the average EPSC amplitude per minute and normalized to baseline.

For NMDA/AMPA ratio measurement, cells were first held at − 70 mV and recorded AMPA-EPSCs for 2 mins at the frequency of 0.1 Hz, and then the holding potential was gradually increased to + 40 mV. NMDA-EPSCs were recorded for 6 sweeps per minute for 5 mins. The AMPA current was defined by measuring the amplitudes of NMDA-EPSCs (+ 40 mV) at the peak time of AMPA-EPSCs (− 70 mV). The NMDA current was defined by measuring the amplitudes of NMDA-EPSCs 50 ms after EPSCs onset.

The field recordings were performed under current-clamp mode with the recording electrodes (3–5 MΩ) filled with ACSF, and a bipolar metal stimulating electrode was used to evoke field excitatory postsynaptic potential (fEPSP). Field LTP was induced by Theta-burst (TBS) containing six episodes with an interval of 10 s. Each episode of TBS comprised five bursts at 5 Hz, with each burst composed of five pulses at 100 Hz. fEPSP recordings were conducted for 10 mins for baseline and 40 mins after induction at the frequency of 0.1 Hz. The fEPSP slope was analyzed to measure the field LTP.

All data were collected online using Patchmaster software (Heka instruments) sampled at 10 kHz, low-pass filtered at 2 kHz, and analyzed offline by Fitmaster (Heka instruments) or Clampfit10 (Molecular Devices).

#### Biocytin-filled neuron staining

After whole-cell recording, slices were fixed in 4% paraformaldehyde for 48 h at 4 °C and then permeabilized with 0.5% Triton X-100 for 30 mins. Slices were then incubated with Atto 550-streptavidin (Sigma, 96,404, 1:500) in 0.5% Triton X-100 + 1% BSA in 0.02 M PBS at room temperature for 2 h and then washed with PBS twice. Slices were then mounted on slides and coverslipped with glycerin mounting medium. Images of dendrites were captured under a 20x object lens and spines were captured under a 100x oil lens with a confocal microscope (Olympus, FV1000) in Z-stack mode.

For co-labeling of biocytin and YFP, slices were fixed and permeabilized and then blocked with 10% serum in 0.1 M PBS for 2 h at room temperature. Chicken anti-GFP (Abcam, ab13970, 1:500) was applied at 4 °C overnight, followed by incubation of goat anti-chicken 488 (Molecular Probes, A11039, 1:500) secondary antibody. Atto 550-streptavidin was then applied as described above.

### Golgi staining and morphometric analysis

Golgi-cox staining was performed using the FD Rapid Golgi Stain Kit (FD Neurotechnologies). After dissection, brains were immersed in the mixture of solution A and B in the dark at room temperature for 14 days. Brains were then transferred to solution C and immersed for 7 days. Slices were sectioned at 120 μm with a vibrating microtome (Leica Microsystems, VT1000s) and stained following the manufacturer’s guide. Hippocampal neuron images were captured under Z-stack mode using an EVOS FL auto microscope (Life technology) with a 20x object lens for dendritic analysis and a 40x object lens for spines. Dendrites were traced manually using the NeuronJ plugin in ImageJ. Sholl analysis was applied to assess dendrite complexity by measuring the dendritic intersections in concentric circles per 10 μm from the cell soma. Significance was determined by a two-way repeated-measures analysis of variance (RM 2-ANOVA; genotype and circle radius as factors). The analysis of dendritic spine density was performed by calculating spines on the first 50 μm of the apical secondary branch proximate to the cell soma.

### Primary neuron culture and immunostaining

Primary hippocampal neurons were prepared from E16.5 *Nex-Cre*;*Foxg1*^*fl/fl*^ and *Foxg1*^*fl/fl*^ pups. Hippocampi were dissected in ice-cold HBSS (Thermo Fisher Scientific, 14,170,112) and digested with 0.125% trypsin (Thermo Fisher Scientific, 25,200) for 8 mins at 37 °C. Trypsin was then neutralized by serum containing DMEM (Thermo Fisher Scientific, 11,330,032) and removed by centrifugation at 1000 rpm for 5 mins. Neurons were then suspended and plated at a density of 2 × 10^4^ cells/cm^2^ on poly-L-lysine coated glass coverslips in neurobasal A medium (Thermo Fisher Scientific, 10,888,022) supplemented with 2% B27 (Thermo Fisher Scientific, 17,504,044), 1% GlutaMax-1 (Thermo Fisher Scientific, 17,504,044) and 0.2% penicillin/streptomycin (Thermo Fisher Scientific, 15,070,063).

For morphological analysis, neurons were fixed with 4% paraformaldehyde at 37 °C for 15 mins. After fixation, neurons were permeabilized with 0.1% Triton X-100 for 15 mins and blocked with 10% normal goat serum in PBS for 1 h at room temperature. Neurons were incubated with primary antibodies at 4 °C overnight in the dark, followed by incubation with secondary fluorophore-conjugated antibodies. For axonal analysis, rabbit anti-Tau (Abcam, ab64193, 1:500) was applied at DIV 5, and mouse anti-Map 2 (Chemicon, MAB378, 1:1000) was applied at DIV 7 and 14 for dendritic analysis. Secondary antibodies were donkey anti-rabbit 555 (1:500, Molecular Probes, A31572) and donkey anti-mouse 550 (1:500, Thermo Fisher Scientific, SA5–10067). Neuron images were captured under Z-stack mode using an EVOS FL auto microscope (Life technology). Dendrites and axons were traced and analyzed by the same method used for the Golgi staining.

### Statistics

All data were presented as the mean ± standard error of the mean (SEM) and were analyzed using the software GraphPad Prism 5.0 (GraphPad Software) and OriginPro 8 (OriginLab Corp). Student’s *t*-test was applied to determine the significance between two groups. One-way ANOVA with the post hoc Tukey test was used to analyze the time in each chamber in the social behavior test and the time in quadrants in the Morris-water maze. Two-way ANOVA was applied to compare LTP, dendritic complexity, the learning curve in the Morris-water maze and the fear curve in the contextual and cued fear conditioning test. Statistical significance was defined at *P* < 0.05 and presented as *P < 0.05, **P < 0.01, ***P < 0.001, ****P < 0.0001.

## Results

### Loss of *Foxg1* leads to deficiency in social and cognitive behaviors

**Fig. 1:**
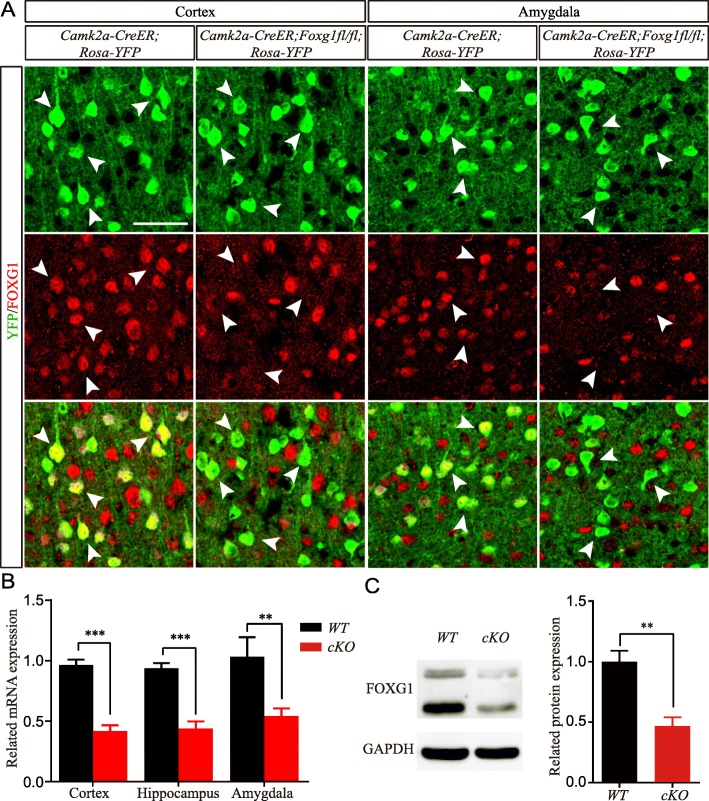
*Foxg1* is efficiently eliminated in cKO mice. **a** Immunostaining for anti-FOXG1 with YFP in the adult cortex and the amygdala, showing that FOXG1 was effectively deleted. **b** *Foxg1* mRNA level was greatly reduced in the cortex, the hippocampus and the amygdala (Cortex, *P* = 0.0002; Hippocampus, *P* = 0.0005; WT, *n* = 4; cKO, *n* = 4; Amygdala, *P* = 0.0089, WT, *n* = 3; cKO, n = 3; *t* test). **c** Hippocampal FOXG1 protein level was greatly reduced via Western blot (WT, *n* = 4; cKO, *n* = 3, *P* = 0.0073). **P < 0.01, ***P < 0.001. Scale bar, 100 μm

Patients suffering from *FOXG1* syndrome exhibit mental retardation, absence of social ability and intellectual disabilities [21]. To explore the cellular basis underlying the symptoms and to investigate the function of *Foxg1* in telencephalic mature neurons, *Foxg1* was deleted by crossing *Foxg1*^*fl/fl*^ with a *Camk2a-Cre*^*ER*^ line combined with tamoxifen induction. Tamoxifen was administered five times around P60. Thirty days later, brains were collected for analysis. A reporter line *Rosa-YFP* was introduced to label neurons in which Cre-mediated recombination occurred. As shown in Fig. 1a, FOXG1 was efficiently disrupted in YFP^+^ cells. FOXG1 was strongly expressed in YFP^+^ neurons in the *Camk2a-Cre*^*ER*^*;Rosa-YFP* control mice, while in *Camk2a-Cre*^*ER*^*;Foxg1*^*fl/fl*^*;Rosa-YFP* (cKO) neurons, FOXG1 was almost undetectable. The FOXG1^+^YFP^−^ cells are neurons in which Cre-mediated recombination did not occur and the proportion was about 40%. We found that the total mRNA level was significantly decreased in the *Camk2a-Cre*^*ER*^*;Foxg1*^*fl/fl*^*;Rosa-YFP* cortex, hippocampus and amygdala compared with wide-type (WT) (Fig. 1b), and the total protein level was reduced 60% in the *Camk2a-Cre*^*ER*^*;Foxg1*^*fl/fl*^*;Rosa-YFP* hippocampus compared with WT (Fig. 1c).

**Fig. 2:**
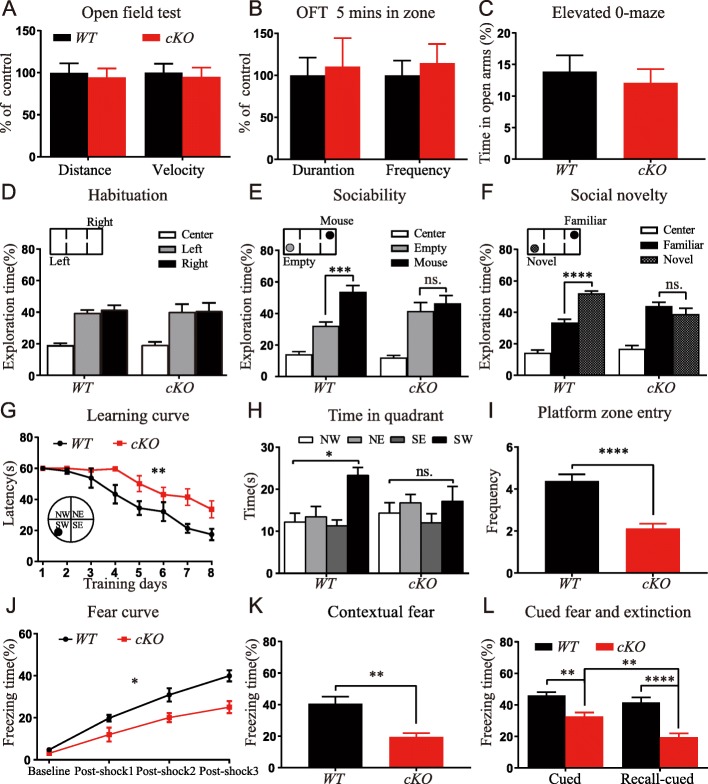
Disruption of *Foxg1* results in impaired social abilities, learning and memory. **a** Average velocity and total distance moved in 30 mins were similar between WT and cKO mice during open-field test (*P* = 0.76; *t* test). **b** The duration and frequency in the center zone during the first 5 mins in open-field test were indistinguishable (*P* = 0.61). **c** Comparable open-arm time in elevated O-maze (*P* = 0.65). **d** Mice showed no preference to the two lateral chambers in the habituation phase of the social behavior test. (WT, Left vs. Right, q = 0.96; cKO, Left vs. Right, q = 0.15; one-way ANOVA with post hoc Tukey test). **e** cKO mice had no preference during the social recognition phase (WT, Empty vs. Mice, q = 6.606; cKO, Empty vs. Mice, q = 1.175). **f** cKO mice did not show any preference during the social novelty phase (WT, Familiar vs. Novel, q = 9.033; cKO, Familiar vs. Novel, q = 0.4486). **g** cKO mice spent more time finding the hidden platform during the training sessions in the Morris water maze (F (7, 98) = 2.92, *P* = 0.0081; two-way ANOVA*,* time x group, repeated measure). **h** WT mice spent significantly more time in the target quadrant, while cKO mice did not search selectively (WT, F (2.122, 14.85) = 7.73, *P* = 0.0045; cKO, F (1.973, 13.81) = 0.85, *P* = 0.45; one-way ANOVA). **i** cKO mice showed less platform zone entry (*P* = 0.0003). **j** cKO mice exhibited decreased freezing time during fear conditioning training (F (3, 21) = 4.363, *P* = 0.015; two-way ANOVA). **k** cKO mice exhibited decreased freezing time during the contextual fear conditioning test (*P* = 0.001). **l** cKO mice showed less freezing time during the cued fear conditioning test (*P* = 0.001) and cued fear memory recall test (WT vs. cKO, *P* < 0.0001; cKO, Cued vs. Cued-recall, *P* = 0.0016;). WT, *n* = 8; cKO, *n* = 8. *P < 0.05, **P < 0.01, ***P < 0.001, ****P < 0.0001

To investigate the impact of *Foxg1* deletion on cognitive, social, and emotional behaviors, an open-field test was first performed. As shown in Fig. 2a, the total distance and the mean velocity within a 30 min duration were undistinguishable between cKO and WT, indicating that locomotor activity was unaffected. Considering that the behavior within the first 5 mins best reflects the level of anxiety, we measured the duration spent in the center zone during the first 5 mins. The time spent in the center zone and the frequency of entering the center zone were comparable in WT and cKO mice (Fig. 2b), indicating that the anxiety level was unchanged. When *Foxg1* cKO mice were subjected to the elevated-O maze test, an identical result was obtained (Fig. 2c). To evaluate social ability, we performed a three-chamber test. Both WT and cKO mice displayed no preference to the two empty chambers during the habituation phase (Fig. 2d). However, during the social recognition phase, WT mice spent more time around the chamber caged with a mouse, while cKO mice had no preference (Fig. 2e). In the social novelty test, WT mice displayed a preference for the chamber with a novel mouse, while cKO mice had no preference (Fig. 2f). These data indicate that disruption of *Foxg1* results in deficient social ability but has no effect on anxious behavior.

To assess spatial learning and memory, we then performed a Morris water maze test. During the acquisition phase, the latency of finding the hidden platform for both genotypes was progressively reduced, but cKO mice spent more time searching for the platform during the 8 day-training session (Fig. 2g), indicating a significant defect in spatial learning ability. In the probe trial which was performed 24-h later, WT mice spent significantly more time in the target quadrant and showed a high crossing frequency over the zone where the platform was previously located. However, the cKO mice did not exhibit any preference to the four quadrants and showed a lower frequency (Fig. 2h-i), indicating that the cKO mice had diminished spatial memory acquisition and memory recall. We also performed a contextual and cued fear conditioning test. During the training phase, mice were given a 30 s sound stimulation followed by an electric foot shock. cKO mice showed decreased freezing time compared with WT mice, indicating that fear learning was impaired (Fig. 2j). Contextual and cued memory were then separately tested in the following 2 days. The freezing time in both the contextual and cued conditioning test was significantly reduced in cKO mice compared with WT mice (Fig. 2k-l). Moreover, the freezing time of the cKO mice was greatly reduced in the cued fear memory recall compared with WT mice and with it was in the cued condition test, whereas WT mice did not show a reduction (Fig. 2l). Thus, disruption of *Foxg1* results in defective fear memory and accelerate memory loss.

### *Foxg1* removal impairs hippocampal long-term potentiation

**Fig. 3:**
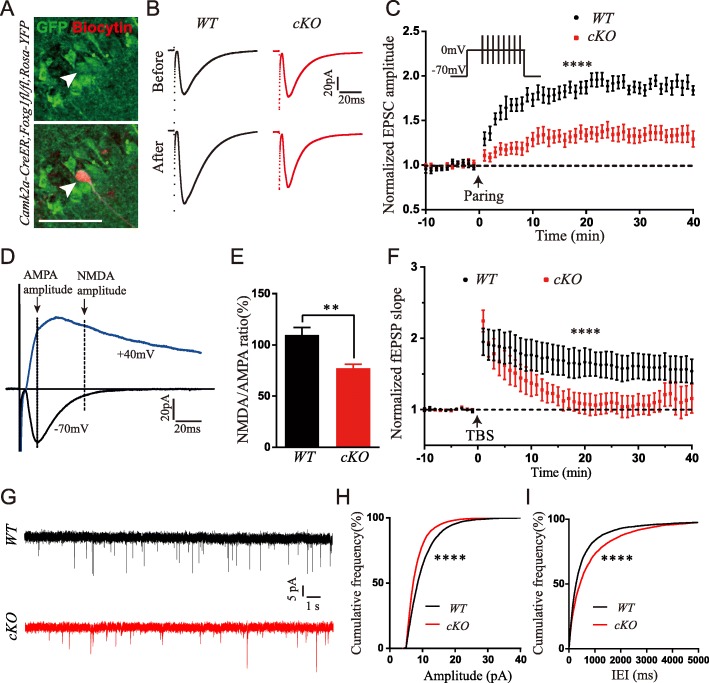
Hippocampal LTP, NMDA receptor activity and synaptic transmission were reduced. **a** The overlap of post hoc staining of anti-YFP and biocytin verified the precise recording of *Foxg1*-deficient neurons (Arrow head: upper, YFP+ cells; lower, YFP overlap with biocytin staining. **b** EPSC amplitude was obviously increased in WT but not cKO mice after paring induction in whole-cell recording (Upper panel, EPSC traces before induction; lower panel, EPSC traces after induction). **c** LTP was reduced in cKO mice (WT, 15 cells from 6 mice; cKO, 19 cells from 6 mice, F (50, 1632) = 5.021, *P* < 0.0001; two-way ANOVA, time x group, repeated measure). **d** Representative traces of AMPA-EPSC (+ 40 mV) and NMDA-EPSC (− 70 mV) from whole-cell recording. **e** NMDA to AMPA ratio was decreased in cKO mice (WT, 17 cells from 6 mice; cKO, 10 cells from 6 mice, *P* = 0.0035; *t* test). **f** Recordings of field EPSP showed a decreased LTP after TBS induction in cKO mice (WT, 6 slices from 3 mice; cKO, 5 slices from 3 mice, F (43, 387) = 3.657, *P* < 0.0001, two-way ANOVA, time x group, repeated measure). **g** Sample traces of spontaneous EPSCs (sEPSC) recorded from hippocampus CA1 pyramidal neurons of WT and cKO. **h-i** The amplitude was decreased whereas the inter-event intervals were increased in cKO neurons (WT, 33 cells from 6 mice, cKO, 25 cells from 6 mice. Amplitude, *P* < 0.0001; IEI, *P* < 0.0001, K-S test). Scale bar, 50 μm. **P < 0.01, ****P < 0.0001

To verify whether hippocampal LTP contributed to the impaired learning and memory in cKO mice, we performed whole-cell recordings in acute hippocampal slices. Evoked excitatory postsynaptic current (eEPSC) was recorded in the Schaffer collateral pathway, and LTP was induced by paring [28]. The *Rosa-YFP* line was introduced to label hippocampal neurons (Fig. 3a). The eEPSC amplitude after paring induction in cKO neurons barely increased, in contrast to WT mice where it was significantly increased (Fig. 3b). LTP was significantly reduced in cKO compared to WT mice (Fig. 3c). We then investigated the effect of *Foxg1* disruption on the NMDA receptor, which is known to be critical for LTP maintenance [29]. The activity of the NMDA receptor was measured by normalized evoked NMDA to AMPA current as previously reported (Fig. 3d) [30, 31]. We found that the NMDA/AMPA ratio was reduced by approximately 29.6% in cKO mice (Fig. 3e). To further verify the LTP defects, we applied field recording and LTP was induced by Theta-burst. By measuring the field excitatory postsynaptic potential (fEPSP) slope, we found that the LTP was also significantly decreased in cKO mice (Fig. 3f), coincident with the result obtained by whole-cell recording. To further study the impact of *Foxg1* deletion on the basic synaptic transmission, we analyzed the spontaneous excitatory postsynaptic currents (sEPSC) of CA1 pyramidal neurons. We found that the amplitude of the sEPSCs was significantly decreased, whereas the inter-event interval (IEI) was remarkably increased in cKO mice (Fig. 3g-i). These results suggest that disruption of *Foxg1* led to abnormal synaptic transmission, reduced NMDA receptor activity and finally resulted in the impairment of the hippocampal LTP.

### Reduced dendritic complexity and spine density after *Foxg1* ablation

**Fig. 4:**
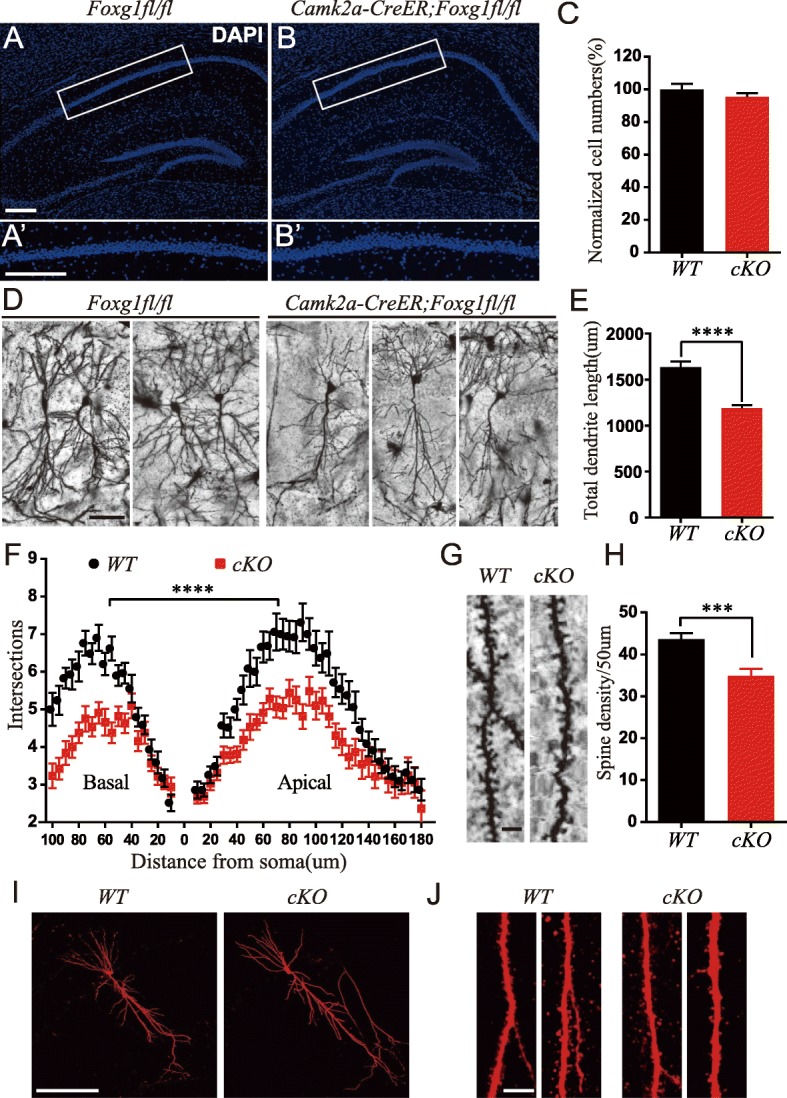
*Foxg1* elimination led to reduced dendritic complexity and spine density. **(a-b′**) Undetectable changes in hippocampal structure between WT and cKO viewed by DAPI staining (**a’** and **b′**, High magnification views of boxed CA1 regions in A and B, respectively. Scale bar, 200 μm). **c** Cell counting of boxed CA1 regions in A’ and B′ showed comparable cell numbers (WT, 6 slices from 3 mice; cKO, 6 slices from 3 mice, *P* = 0.2795; *t* test). **d** cKO mice displayed an obvious reduction in dendritic branches in hippocampal CA1 pyramidal neurons as viewed by Golgi staining (Scale bar, 50 μm). **e** Total dendritic length was significantly reduced in cKO mice (WT, 37 cells from 4 mice; cKO, 25 cells from 4 mice, *P* < 0.0001; *t* test). **f** cKO mice exhibited reduced dendritic complexity in both apical and basal dendrites of hippocampal CA1 pyramidal neurons (WT, 35 cells from 4 mice; cKO, 32 cells from 4 mice, F (54, 3575) = 2.568, *P* < 0.0001; two-way ANOVA, distance x group, repeated measure). **g** Representative images of spines from apical secondary branches (Scale bar, 2.5 μm). **h** Spine density was significantly reduced in cKO mice (WT, 54 cells from 4 mice; cKO, 35 cells from 4 mice, *P* = 0.0002; *t* test). **i-j** Post hoc staining of biocytin showed a strong reduction in dendritic branches (**i**) and spines (**j**) in cKO neurons (Scale bars, **i**: 200 μm; **j**: 20 μm). ***P < 0.001, ****P < 0.0001

To explore the cellular basis underlying the reduction of LTP, we then examined whether there was any cell loss after *Foxg1* ablation. The numbers of pyramidal neurons in the CA1 area were compared, and there were no obvious changes detected (Fig. 4a-c). Immunostaining for anti-Caspase3 showed no increase in apoptosis in cKO mice (data not shown). We next performed Golgi staining to examine the morphology of pyramidal neurons. As shown in Fig. 4d, we observed a remarkable reduction in both basal and apical dendritic complexity in cKO neurons. The dendritic complexity was then quantified by counting the intersections where dendrites crossed concentric circles drawn at 10-μm intervals around the neuronal cell bodies. Statistical analysis showed an obvious decrease in the number of intersections in cKO compared with WT neurons. The total dendritic length was reduced by approximately 27.2% (Fig. 4d-f). We also examined the spine numbers on the first 50 μm of an apical secondary dendritic branch of pyramidal neurons and found a significant decrease (Fig. 4g-h). Further evidence from post hoc staining of biocytin after whole cell recording showed similar results (Fig. 4i-j).

### Ablation of *Foxg1* impairs dendritic and axonal growth in vitro

**Fig. 5:**
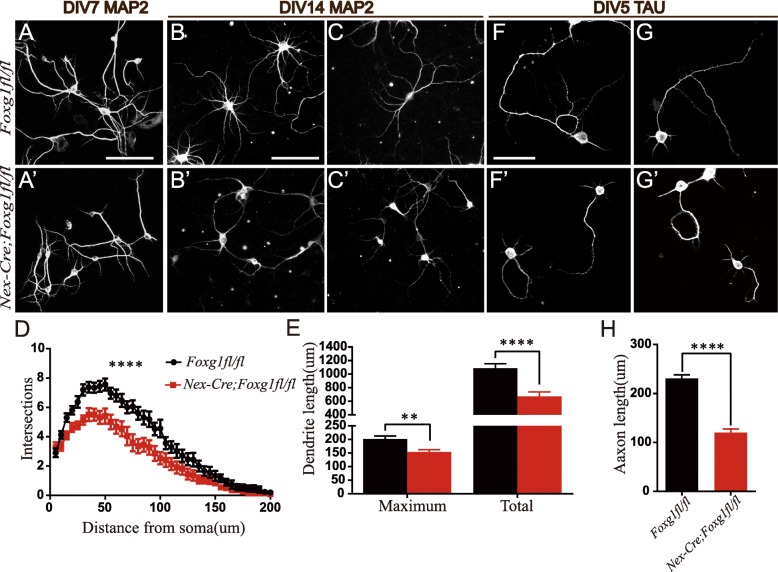
Loss of *Foxg1* led to impairment in dendritic complexity and axonal maintenance. **(a-c′)** Immunostaining of anti-Map 2 in cultured primary neurons at DIV 7 (**a, a’**) and DIV 14 (**b-c′**) exhibited decreased dendritic branches in *Foxg1*-deficient neurons (Scale bar, 100 μm). **d** Application of Sholl analysis showed decreased dendritic complexity at DIV 14 in *Foxg1*-deficient neurons (WT *n* = 30, cKO *n* = 22, F (39, 1950) = 3.085, *P* < 0.0001; two-way ANOVA, distance x group, repeated measure). **e** Both maximum and total dendritic length were reduced in *Foxg1*-deficient neurons (WT, n = 30; cKO, *n* = 20; Maximum length, *P* = 0.0021; Total length, *P* < 0.0001; *t* test). **f-g’** *Foxg1*-deficient neurons displayed a shorter axon at DIV 5 as viewed by immunostaining of anti-Tau (Scale bar, 50 μm). **h** Quantification of axonal length revealed significantly reduced axonal length in *Foxg1*-deficient neurons (WT, *n* = 57; cKO, *n* = 42, *P* < 0.0001; *t* test). Data was from three repeated cell culture experiments, **P < 0.01, ****P < 0.0001

To further investigate the role of *Foxg1* in dendritic complexity, we conducted primary cell culture. Hippocampi were dissected from E16.5 brains in which *Foxg1* was deleted in postmitotic neurons by crossing *Nex-Cre* with *Foxg1*^*fl/fl*^ and neurons were cultured for 7 or 14 days. Neurons were then stained with anti-Map 2 to view dendritic development. As shown in Fig. 5a-c’, compared with WT, the dendritic branches were severely decreased at DIV7 in *Nex-Cre;Foxg1*^*fl/fl*^ neurons (Fig. 5a, a’). Identical results were obtained at DIV14 (Fig. 5b-c’). Statistical analysis showed that the dendritic intersections were significantly decreased in *Nex-Cre;Foxg1*^*fl/fl*^ neurons at DIV14 (Fig. 5d), and the length of the longest dendrite branch and the total length of dendrites were decreased by approximately 24 and 38.3%, respectively, when compared with WT neurons (Fig. 5e). We also investigated the development of axons by immunostaining for anti-Tau at DIV5. WT neurons displayed a long well-developed axon; however, the axon in cKO neurons was remarkably shorter (Fig. 5f-g). The axonal length was decreased by 47.9% compared with that of WT (Fig. 5h). Taken together, our data demonstrate that loss of *Foxg1* leads to impairment of dendritic complexity and axonal maintenance.

## Discussion

In this study, we reported the role of *Foxg1* in the maintenance of neural plasticity. Ablation of *Foxg1* in postmitotic/mature neurons led to severely decreased dendritic complexity and spine density that consequently caused impaired synaptic transmission, LTP and NMDA receptor activity, which in turn resulted in social and cognitive behavioral defects. Our results demonstrate an important role for *Foxg1* in dendrite arborization and spine maintenance and provide insights into the cellular basis of *FOXG1* syndrome.

### Ablation of *Foxg1* leads to impairment of learning and memory attributed to the impairment of LTP

Patients with *FOXG1* syndrome suffer from mental retardation, impaired social ability and cognitive deficiency [21]. Embryonic death of the conventional *Foxg1* knockout mice makes it unachievable to explore the postnatal role of *Foxg1* [9, 32]. Here, we took advantage of the *Camk2a-Cre*^*ER*^ and *Nex-Cre* lines to conditionally disrupt *Foxg1* in mature/postmitotic neurons [24, 33]. We demonstrated that *Foxg1* removal led to decreased spatial learning and memory as well as contextual and cued fear conditioning memory. *Foxg1* cKO mice also displayed a deficiency in social behavior but had no effects on locomotor and anxiety related behaviors.

LTP has been extensively shown to underlie the synaptic basis of learning and memory [34, 35]. Abnormal LTP has been detected in a variety of neuronal diseases such as Rett syndrome, Autism Spectrum Disorder (ASD) and Schizophrenia (SCZ) [5, 36–38]. Here, we reported that ablation of *Foxg1* led to impaired hippocampal LTP. Moreover, the activity of postsynaptic NMDA receptors, which is responsible for LTP maintenance [29], was significantly decreased in *Foxg1* cKO mice. A recent study has shown that *Neuronatin (Nnat)* mRNA level was increased in *Foxg1* heterozygotes [39]. *Nnat* was reported to be involved in the calcium signaling and may regulate neuronal excitability and receptor trafficking [40, 41]. Thus, *Foxg1* may affect NMDA receptor activity through regulating *Nnat* expression. The impaired LTP could also attribute to the decreased synaptic transmission which was found in the *Foxg1* cKO mice. Previously, it has been reported that *Foxg1*^+/−^ mice exhibit defective contextual memory attributed to decreased postnatal neurogenesis in the dentate gyrus [22]. Here, our study gave new insight into the cellular basis underlying the impaired cognitive and social behaviors.

### Deletion of *Foxg1* affects dendritic complexity and spine density

It has been reported that abnormalities in the architecture of dendrites and alterations in spine number are closely associated with changes in LTP and impaired social and cognitive abilities, which are observed in many neuronal disorders [4]. Brancaccio et al. demonstrated that overexpression of *Foxg1* in neuronal-restricted progenitors promotes neurite outgrowth; on the contrary, haploinsufficiency of *Foxg1* in progenitors leads to poorer neurite morphologies [42]. Recently, overexpression and knockdown of *Foxg1* in postmitotic neurons revealed that *Foxg1* promotes neurite elongation [23]. In our previous studies, we demonstrated that *Foxg1* is required for neurite development in cortical interneurons [14, 25]. Here, we showed that removal of *Foxg1* in adulthood resulted in decreased dendritic complexity, further confirming the role of *Foxg1* in the development and maintenance of dendritic arborization. Furthermore, we found that spine density was also obviously reduced, which may lead to the reduction of synaptic transmission. Our study revealed that *Foxg1* regulated dendritic complexity in mature neurons and is critical for maintaining spine density. Thus, deficiency in neurites led to the impairment of hippocampal LTP and, in turn, abnormal social and cognitive behaviors.

## Data Availability

The data generated or analyzed during this study are included in this published article.

## References

[1] Arikkath J (2012). Molecular mechanisms of dendrite morphogenesis. Front Cell Neurosci.

[2] Emoto K (2011). Dendrite remodeling in development and disease. Develop Growth Differ.

[3] Koleske AJ (2013). Molecular mechanisms of dendrite stability. Nat Rev Neurosci.

[4] Penzes P, Cahill ME, Jones KA, VanLeeuwen JE, Woolfrey KM (2011). Dendritic spine pathology in neuropsychiatric disorders. Nat Neurosci.

[5] Xu X, Miller EC, Pozzo-Miller L (2014). Dendritic spine dysgenesis in Rett syndrome. Front Neuroanat.

[6] Selemon LD, Goldman-Rakic PS (1999). The reduced neuropil hypothesis: a circuit based model of schizophrenia. Biol Psychiatry.

[7] Hutsler JJ, Zhang H (2010). Increased dendritic spine densities on cortical projection neurons in autism spectrum disorders. Brain Res.

[8] Tao W, Lai E (1992). Telencephalon-restricted expression of BF-1, a new member of the HNF-3/fork head gene family, in the developing rat brain. Neuron.

[9] Xuan S, Baptista CA, Balas G, Tao W, Soares VC, Lai E (1995). Winged helix transcription factor BF-1 is essential for the development of the cerebral hemispheres. Neuron.

[10] Hanashima C, Li SC, Shen L, Lai E, Fishell G (2004). Foxg1 suppresses early cortical cell fate. Science.

[11] Tian C, Gong Y, Yang Y, Shen W, Wang K, Liu J (2012). Foxg1 has an essential role in postnatal development of the dentate gyrus. J Neurosci.

[12] Miyoshi G, Fishell G (2012). Dynamic FoxG1 expression coordinates the integration of multipolar pyramidal neuron precursors into the cortical plate. Neuron.

[13] Kumamoto T, Hanashima C (2017). Evolutionary conservation and conversion of Foxg1 function in brain development. Develop Growth Differ.

[14] Yang Y, Shen W, Ni Y, Su Y, Yang Z, Zhao C (2017). Impaired interneuron development after Foxg1 disruption. Cereb Cortex.

[15] Han X, Gu X, Zhang Q, Wang Q, Cheng Y, Pleasure SJ (2018). FoxG1 directly represses dentate granule cell fate during forebrain development. Front Cell Neurosci.

[16] Du A, Wu X, Chen H, Bai QR, Han X, Liu B, et al. Foxg1 directly represses Dbx1 to confine the POA and subsequently regulate ventral Telencephalic patterning. Cereb Cortex. 2019. 10.1093/cercor/bhz037. [Epub ahead of print] 10.1093/cercor/bhz037 30843579

[17] Pratt DW, Warner JV, Williams MG (2013). Genotyping FOXG1 mutations in patients with clinical evidence of the FOXG1 syndrome. Mol Syndromology.

[18] Shoichet SA, Kunde SA, Viertel P, Schell-Apacik C, von Voss H, Tommerup N (2005). Haploinsufficiency of novel FOXG1B variants in a patient with severe mental retardation, brain malformations and microcephaly. Hum Genet.

[19] Ariani F, Hayek G, Rondinella D, Artuso R, Mencarelli MA, Spanhol-Rosseto A (2008). FOXG1 is responsible for the congenital variant of Rett syndrome. Am J Hum Genet.

[20] Le Guen T, Bahi-Buisson N, Nectoux J, Boddaert N, Fichou Y, Diebold B (2011). A FOXG1 mutation in a boy with congenital variant of Rett syndrome. Neurogenetics.

[21] Kortum F., Das S., Flindt M., Morris-Rosendahl D. J., Stefanova I., Goldstein A., Horn D., Klopocki E., Kluger G., Martin P., Rauch A., Roumer A., Saitta S., Walsh L. E., Wieczorek D., Uyanik G., Kutsche K., Dobyns W. B. (2011). The core FOXG1 syndrome phenotype consists of postnatal microcephaly, severe mental retardation, absent language, dyskinesia, and corpus callosum hypogenesis. Journal of Medical Genetics.

[22] Shen L, Nam HS, Song P, Moore H, Anderson SA (2006). FoxG1 haploinsufficiency results in impaired neurogenesis in the postnatal hippocampus and contextual memory deficits. Hippocampus.

[23] Chiola Simone, Do Mihn Duc, Centrone Lucy, Mallamaci Antonello (2018). Foxg1 Overexpression in Neocortical Pyramids Stimulates Dendrite Elongation Via Hes1 and pCreb1 Upregulation. Cerebral Cortex.

[24] Goebbels S, Bormuth I, Bode U, Hermanson O, Schwab MH, Nave KA (2006). Genetic targeting of principal neurons in neocortex and hippocampus of NEX-Cre mice. Genesis.

[25] Shen Wei, Ba Ru, Su Yan, Ni Yang, Chen Dongsheng, Xie Wei, Pleasure Samuel J, Zhao Chunjie (2018). Foxg1 Regulates the Postnatal Development of Cortical Interneurons. Cerebral Cortex.

[26] Wu X, Gu X, Han X, Du A, Jiang Y, Zhang X (2014). A novel function for Foxm1 in Interkinetic nuclear migration in the developing telencephalon and anxiety-related behavior. J Neurosci.

[27] Vorhees CV, Williams MT (2006). Morris water maze: procedures for assessing spatial and related forms of learning and memory. Nat Protoc.

[28] Chen HX, Otmakhov N, Lisman J (1999). Requirements for LTP induction by pairing in hippocampal CA1 pyramidal cells. J Neurophysiol.

[29] Nakazawa K, McHugh TJ, Wilson MA, Tonegawa S (2004). NMDA receptors, place cells and hippocampal spatial memory. Nat Rev Neurosci.

[30] Myme CIO, Sugino K, Turrigiano GG, Nelson SB (2003). The NMDA-to-AMPA ratio at synapses onto layer 2/3 pyramidal neurons is conserved across prefrontal and visual cortices. J Neurophysiol.

[31] Tozzi A, Sclip A, Tantucci M, de Iure A, Ghiglieri V, Costa C (2015). Region- and age-dependent reductions of hippocampal long-term potentiation and NMDA to AMPA ratio in a genetic model of Alzheimer's disease. Neurobiol Aging.

[32] Hanashima C, Shen L, Li SC, Lai E (2002). Brain Factor-1 controls the proliferation and differentiation of neocortical progenitor cells through independent mechanisms. J Neurosci.

[33] Madisen L, Zwingman TA, Sunkin SM, Oh SW, Zariwala HA, Gu H (2010). A robust and high-throughput Cre reporting and characterization system for the whole mouse brain. Nat Neurosci.

[34] Bliss TV, Collingridge GL (1993). A synaptic model of memory: long-term potentiation in the hippocampus. Nature.

[35] Bliss TV, Collingridge GL, Morris RG (2014). Synaptic plasticity in health and disease: introduction and overview. Philos Trans R Soc Lond Ser B Biol Sci.

[36] Moretti P, Levenson JM, Battaglia F, Atkinson R, Teague R, Antalffy B (2006). Learning and memory and synaptic plasticity are impaired in a mouse model of Rett syndrome. J Neurosci.

[37] Savanthrapadian S, Wolff AR, Logan BJ, Eckert MJ, Bilkey DK, Abraham WC (2013). Enhanced hippocampal neuronal excitability and LTP persistence associated with reduced behavioral flexibility in the maternal immune activation model of schizophrenia. Hippocampus.

[38] Jaramillo TC, Speed HE, Xuan Z, Reimers JM, Liu S, Powell CM (2016). Altered striatal synaptic function and abnormal behaviour in Shank3 Exon4-9 deletion mouse model of autism. Autism Res.

[39] Frullanti E, Amabile S, Lolli MG, Bartolini A, Livide G, Landucci E (2016). Altered expression of neuropeptides in FoxG1-null heterozygous mutant mice. EurJ Hum Genet.

[40] Oyang EL, Davidson BC, Lee W, Poon MM (2011). Functional characterization of the dendritically localized mRNA neuronatin in hippocampal neurons. PLoS One.

[41] Sharma J, Mukherjee D, Rao SN, Iyengar S, Shankar SK, Satishchandra P (2013). Neuronatin-mediated aberrant calcium signaling and endoplasmic reticulum stress underlie neuropathology in Lafora disease. J Biol Chem.

[42] Brancaccio M, Pivetta C, Granzotto M, Filippis C, Mallamaci A (2010). Emx2 and Foxg1 inhibit gliogenesis and promote neuronogenesis. Stem Cells.

